# Rehabilitating a severely worn dentition with removable prosthodontics

**DOI:** 10.1038/s41415-023-5583-5

**Published:** 2023-03-24

**Authors:** Stephanie Hackett, Richard Newton, Rahat Ali

**Affiliations:** 41415176006001 Speciality Registrar in Restorative Dentistry, Department of Restorative Dentistry, Liverpool University Dental Hospital, Pembroke Place, Liverpool, L3 5PS, UK; 41415176006002 Consultant in Restorative Dentistry, Department of Restorative Dentistry, Liverpool University Dental Hospital, Pembroke Place, Liverpool, L3 5PS, UK

## Abstract

In the next part of this series on tooth wear management, we discuss the indications and clinical stages for the provision of removable prostheses for the treatment of severely worn and depleted dentitions. The general design features of a complex prosthesis are described for reorganised occlusal schemes and maintenance guidelines are explained. In addition, the clinical stages for three different situations are described: removable-only approaches, and combined fixed and removable in the same arch and separate arches. The value of providing removable prostheses in worn dentitions allows the immediate rehabilitation of severely worn teeth taking a non-invasive and retrievable approach when the remaining dentition is of poor quality or structure and/or there are missing teeth.

## Key points

- Removable prostheses in severely worn dentitions are of value when fixed approaches are not indicated or have low prognosis for success, where there are missing teeth, or as part of a combined fixed-removable treatment plan.
- The general design features of a removable prosthesis include: metal backings on teeth of guarded long-term prognosis; metal or acrylic onlays on posterior teeth where the occlusion allows or vertical dimension is increased; anterior bite platforms where required to gain functional tooth contacts; and minimal apron flanges over teeth used as overdenture abutments.
- Patients who are provided with removable prostheses to manage their tooth wear need to be carefully monitored, with reduced recall intervals of 3-4 months. Close attention should be paid to preventative regimes and identification of new disease.

## Introduction

In this paper, we will discuss the clinical indications for removable prostheses in the treatment of tooth wear. Removable prostheses provide an option for when fixed restorations are contraindicated or have a poor chance of being successful. They can also be provided with fixed restorations as part of a more complex treatment plan. It is the authors' preference to provide definitive cobalt chromium prostheses for all patients, unless a transitional denture is required when acrylic is preferred, for example, in a growing patient, a failing dentition, or as an interim prosthesis during a more protracted fixed-removable treatment plan. Overall, chrome-based dentures offer more rigidity (particularly relevant to parafunctioning patients), tooth support on retained worn teeth, fit more accurately and are less bulky.

In the treatment of tooth wear, removable partial dentures can replace teeth which have been completely lost, or as overdentures when teeth are severely worn. Retaining roots as abutments improves proprioception,^1^ assists with tooth support for the denture, maintains local alveolar bone^2^ and prevents combination syndrome from developing.^3^

**Fig. 1:**
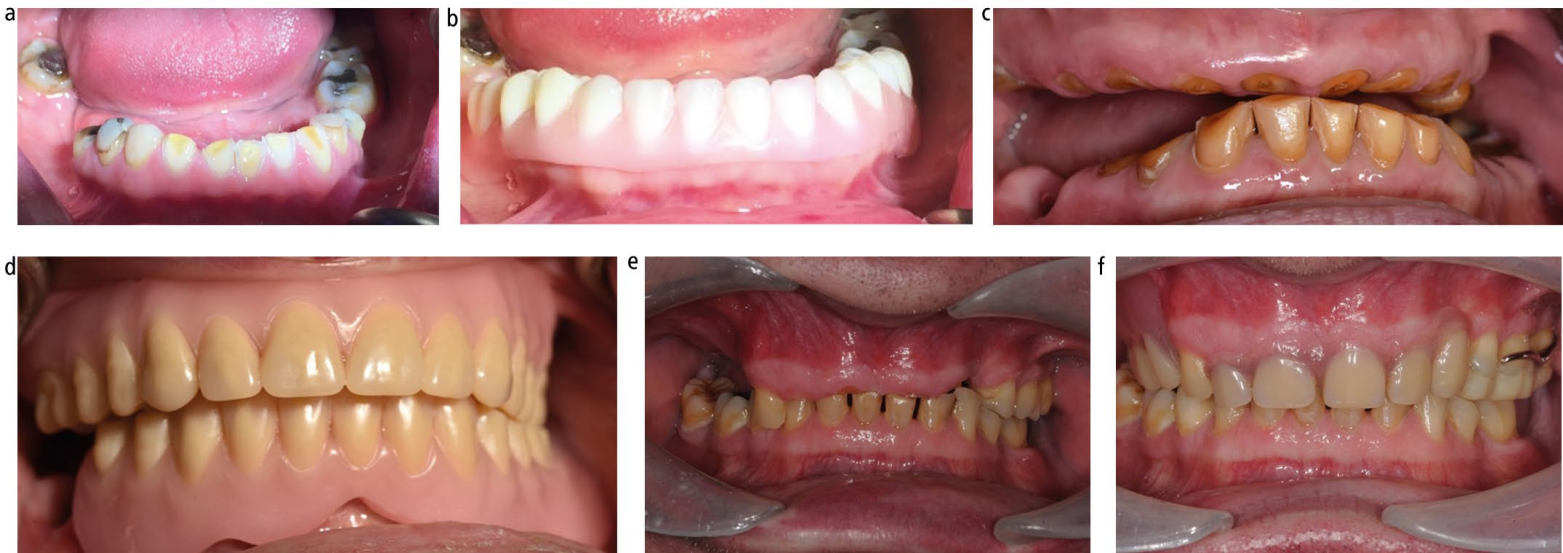
Three broad types of overdentures to treat tooth wear patients. a, b) Onlay denture covers labial surfaces of anterior teeth. c, d) Overdenture covers retained roots of maxillary teeth. e, f) Onlay-overdenture prosthesis covering roots of anterior teeth and onlay rests on posterior teeth

Overdenture-type prostheses can be subdivided in to three main categories (Fig. 1):

- Overdentures - where the denture covers retained roots
- Onlay dentures - components of the denture onlay posterior teeth, similar to a 'table top'. Can re-establish the occlusal plane and restore contacts with the opposing arch
- Overlay dentures - anterior flange and prosthetic teeth cover the labial surfaces of retained anterior teeth whose crowns remain at least partially intact.

Furthermore, partial dentures for the treatment of tooth wear may be provided in any one of the following scenarios:

- Removable partial dentures only
- Fixed restorations and a removable partial denture in the same arch
- Fixed restorations in one arch and a removable prosthesis in the opposing arch.

Studies have shown a high degree of patient satisfaction with overdenture treatment, with over 94% of patients being either mostly or fully satisfied with their prosthesis over several years.^4^^,^^5^ A recent review found that mandibular canine-root-supported overdentures compared favourably with mandibular two-implant supported overdentures.^6^ They found no statistically significant differences with patient satisfaction, prosthodontic complications, or patients' ability to clean their prosthesis.

## Indications for removable prosthodontics

Patients with tooth wear can be managed with fixed or removable treatment options. Should the worn teeth lack adequate tooth structure for bonding or to provide a ferrule effect for a crown, it is not predictable to restore these teeth with a fixed restoration. Also, a history of repeated failure of direct restorations might indicate the lack of potential for bonding or retention on the worn teeth, which might be better used as overdenture abutments. In the authors' experience, teeth which have lost more than 50% coronal tooth structure and have poor quality sclerotic dentine have more frequent failure of direct resin composite build-up treatment. Sclerotic dentine caused by pathological tooth wear has been shown to feature histological obliteration of dentine tubules with intratubular sclerotic casts, in addition to an acid-resistant hypermineralised layer, thus reducing the ability of adhesive systems from achieving the hybrid layer necessary for optimal dentine bonding.^7^ In these cases, consideration could be given to rehabilitate these patients with removable prosthetics. Retaining worn teeth as overdenture abutments affords the benefit of bracing and support for the denture, particularly when there are reduced features of denture bearing anatomy, such as diminutive tuberosities, inadequate sulcus depth and a flat vaulted palate.

**Fig. 2:**
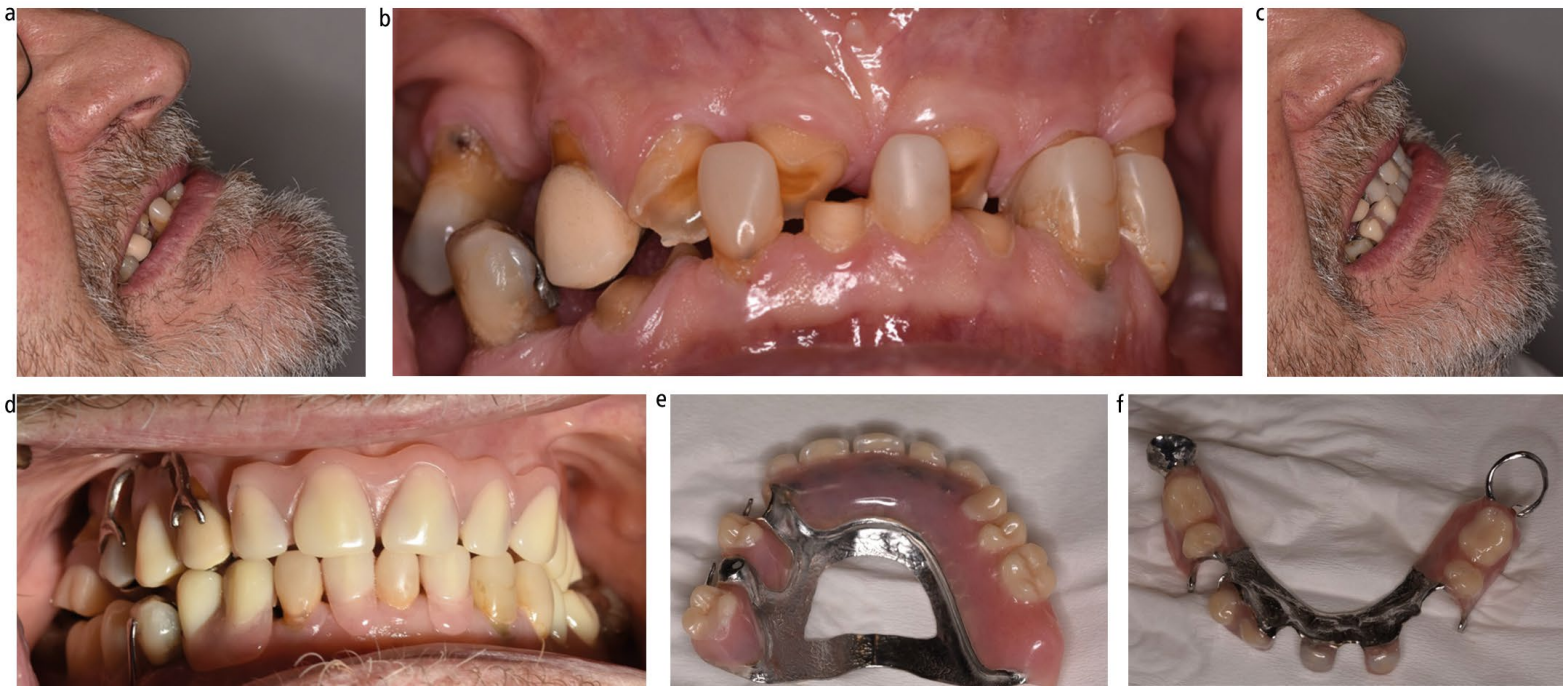
a, b, c, d, e, f) Patient with severe tooth wear and loss of occlusal vertical dimension. Rehabilitated with anterior tooth composites in the mandibular arch, and maxillary and mandibular onlay/overlay partial dentures. Notice how the mandibular prosthesis has metal backings on the lingual surface of the anterior teeth to facilitate easy addition to the denture should these teeth of dubious prognosis be extracted in the future

**Fig. 3:**
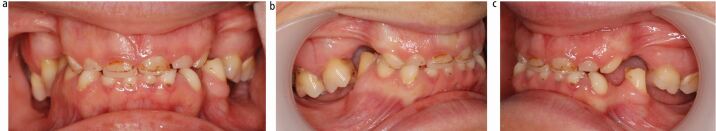
a, b, c) A patient with significant overeruption and dentoalveolar compensation in to opposing edentulous spaces, on a background of localised anterior tooth wear. This patient would benefit from extractions and subsequent resorption of the alveolus to allow prosthetic rehabilitation. Photographs courtesy of Callum Cowan

From an occlusal viewpoint, one should assess the lower face height and decide if the patient is over-closed. If so, this can be restored with an onlay/overlay denture at an increased occlusal vertical dimension (OVD) or by using a retruded contact position that increases the vertical dimension to a suitable level (Fig. 2). Patients with severely worn roots and teeth that have compensated will have a correct lower face height. Such patients, who are unsuitable for surgical crown lengthening, may require extraction and subsequent resorption of the alveolus to create interocclusal space followed by prosthetic replacement of the spaces (Fig. 3).

Assessment and diagnosis of the aetiology of tooth wear will aid in the clinician's determination of fixed restoration prognosis. A bruxist patient with primarily attritional tooth wear will apply excessive force to the teeth and therefore fixed restorations will be at much greater risk of failure.^8^^,^^9^ If a removable alternative is provided, at least it can be removed during episodes of acute parafunction and replaced with a protective guard for sleep to maintain the abutment teeth. The skeletal relationship may further guide the clinician to either fixed or removable prostheses. A Class III incisor relationship (particularly with associated loss of posterior support) may benefit from a maxillary denture, maintaining the worn anterior teeth as overdenture abutments and facilitating the prosthetic incisors into a preferred Class I relationship.

General reasons for providing removable over fixed restorations still apply, including patient-related factors, such as a requirement for reduced clinical time and large edentulous spans not amenable to fixed rehabilitation.

## Benefits of removable prosthodontics

Treatment can be provided reversibly, since the prosthesis is removable and requires minimal tooth preparation. Additionally, the worn dentition with multiple missing posterior teeth may present with reduced vertical facial proportions. A denture can immediately restore this, improving aesthetics, increasing the number of occlusal contacts and therefore restoring function.

**Fig. 4:**
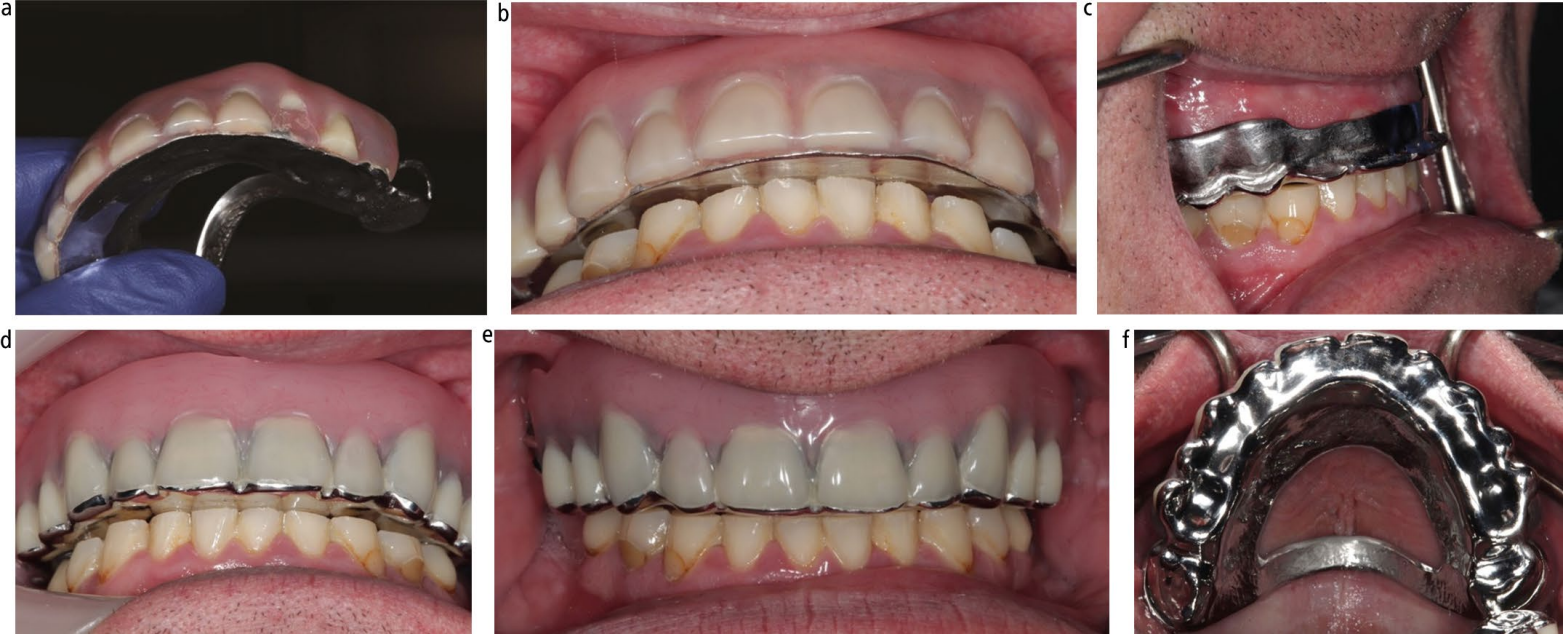
Full coverage overlay-onlay denture to restore worn maxillary teeth. a, b) Old denture was frequently broken as patient was able to break off the prosthetic teeth at the extremities of dynamic occlusion due to severe parafunctioning habits. c, d, e, f) New denture metal framework lips over incisal edge to prevent fracture of the prosthetic teeth in a patient with severe parafunctional tendencies

For patients with primarily attritional tooth wear, chrome dentures can be reinforced with additional metal-based features to resist warpage or breakage of the framework, often seen in severe bruxists (Fig. 2). Clinicians can be creative with these features, such as lipping metal over incisal edges, metal backings on anterior teeth and occlusal onlay rests (Fig. 4).

## Problems with removable prosthodontics

Some patients may not tolerate a partial denture, especially those who gag. We know from existing research that one of the prognostic indictors for success are dentures which replace anterior teeth.^10^ Despite counselling a patient that their partial denture to replace missing posterior teeth will protect their newly provided anterior fixed restorations, it is easy to understand why compliance to wearing a seemingly non-essential prosthesis is still one of the great challenges in the restoration of a worn dentition. Additionally, patients tend to seek a fixed solution over a removable prosthesis for restoration of their worn teeth, as they often associate wearing a denture with negative social stigma. It is down to the clinician to make a balanced judgement on the predictability of fixed and removable treatment, and to guide patients, while attempting to remove some of the negative connotations of removable prostheses.

A removable prosthesis may hinder plaque control.^11^ An onlay/overlay denture can have a more complicated design than usual. It is therefore imperative that patients can demonstrate optimal plaque control and are on a tailored supportive maintenance programme to ensure that the dentures do not act as plaque traps and accelerate the loss of their remaining dentition.^12^

## General design features

**Fig. 5:**
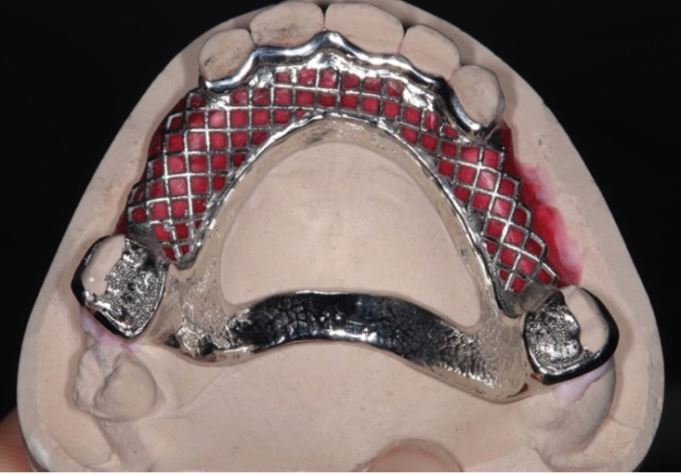
Maxillary partial denture framework with onlay rests on posterior teeth and metal backings on anterior teeth to provide tooth support, indirect retention, and to safeguard the denture against the loss of anterior teath in the future. The denture can be easily modified if an additional tooth is required

We will focus attention on specific denture design principles, which the authors have found to be beneficial when managing worn dentitions. Dentures should be designed to account for loss of further teeth in the future. Teeth with a questionable prognosis should have a (metal) palatal or lingual backing adjacent to it when the denture is seated (Fig. 2, Fig. 5). Should these teeth be lost in the future, an *in situ* impression of the denture can be taken, the backing can be perforated, and replacement acrylic teeth can be added to the existing framework. Backings also benefit from increased tooth support, indirect retention and bracing. However, metal backings placed behind denture teeth can cause grey discolouration of the teeth and it is therefore important to provide a metal-wax-tooth trial for the patient to approve before processing the denture.

**Fig. 6:**
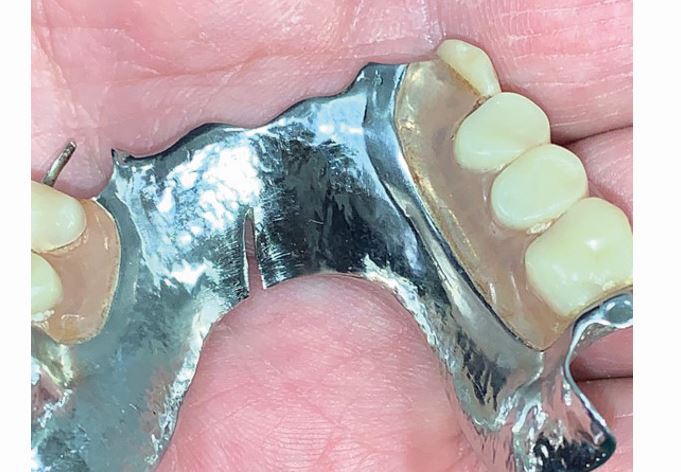
Fracture of chrome denture framework from excessive occlusal forces

Bruxist patients benefit from reinforcement of the denture to prevent warpage. In Figure 4, a patient with a history of broken prosthetic teeth from their first denture was provided with a metal bite platform which lipped over the incisal edges. While this is not the most aesthetic of solutions, it is by far the most functional, and some patients may have to be guided towards this treatment should other treatment fail. U-shaped major connectors are prone to stress and warpage and should be avoided for all patients who parafunction (Fig. 6).

**Fig. 7:**
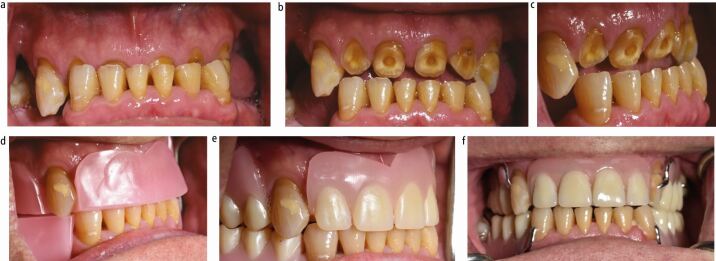
a, b, c, d, e, f) An overclosed patient with loss of posterior occlusal support. On the retruded arc of closure, the first tooth contact offers adequate restorative space to restore the worn anterior teeth

When providing a removable denture for the tooth wear patient, it is necessary to establish whether it is to conform to the existing occlusal vertical dimension, or to be as part of a reorganised occlusal scheme. Generally speaking, the conformative denture is provided following fixed restoration provision in the same arch at a newly established OVD (see later), but the majority of tooth wear patients require reorganisation of the occlusion to account for collapse during tooth wear pathology (Fig. 7). A new vertical dimension is established by components of the denture sitting on teeth or ridges.

The tooth preparation requirements for overdenture abutments are straightforward: removal of sharp edges, undercuts, and unsupported enamel. There is little evidence to support elective endodontic treatment of overdenture abutments,^13^^,^^14^ or coverage of root dentine with glass ionomer cement or other restorative material.^15^ Teeth which will support onlay rests do not require modification; however, should conventional rests be designed, standard protocols for cutting of rest seats is recommended to provide positive seating of the framework. Clinicians should avoid placing rest seats into direct restorations, since repair and maintenance of these restorations in the future is complicated by the metal framework being cast into these areas.

**Fig. 8:**
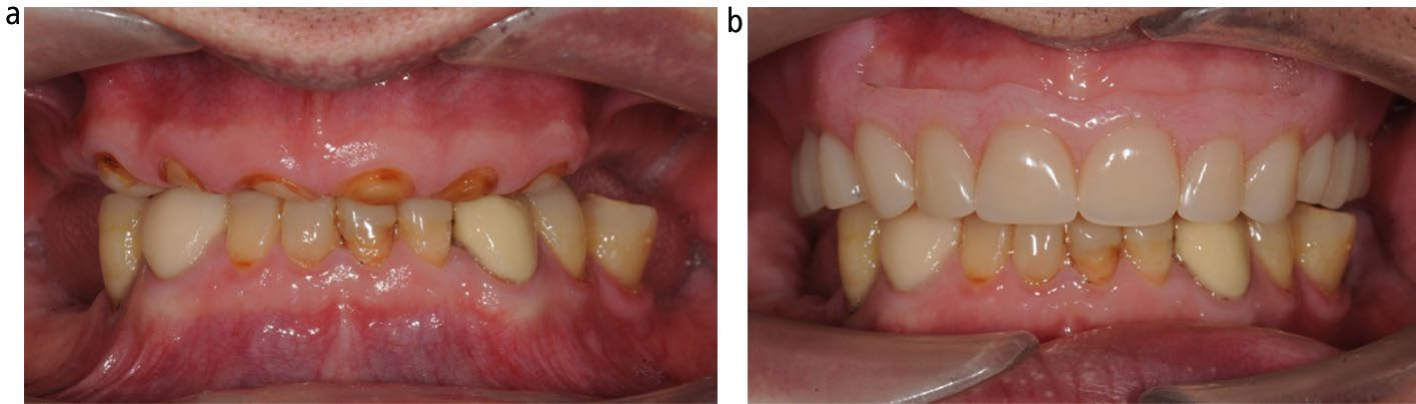
a, b) A partial, or apron flange is useful to minimise overbulking lip support where there are retained roots, whilst aiding in anterior retention of the denture

Lip support is a challenging concept when retaining anterior roots as overdenture abutments. Assessment of the required lip support should be evaluated at the wax-tooth try-in stage to see if increased lip support would be tolerated and suitable. Features such as an apron flange (Fig. 8) not only reduce an excessive lip support but disguise a striking acrylic-soft tissue junction. By surveying the soft tissue undercut on the cast, the flange can be placed just 1 mm beyond the survey line.^16^ This allows future reline or repair of the denture should a root be lost subsequently.

**Fig. 9:**
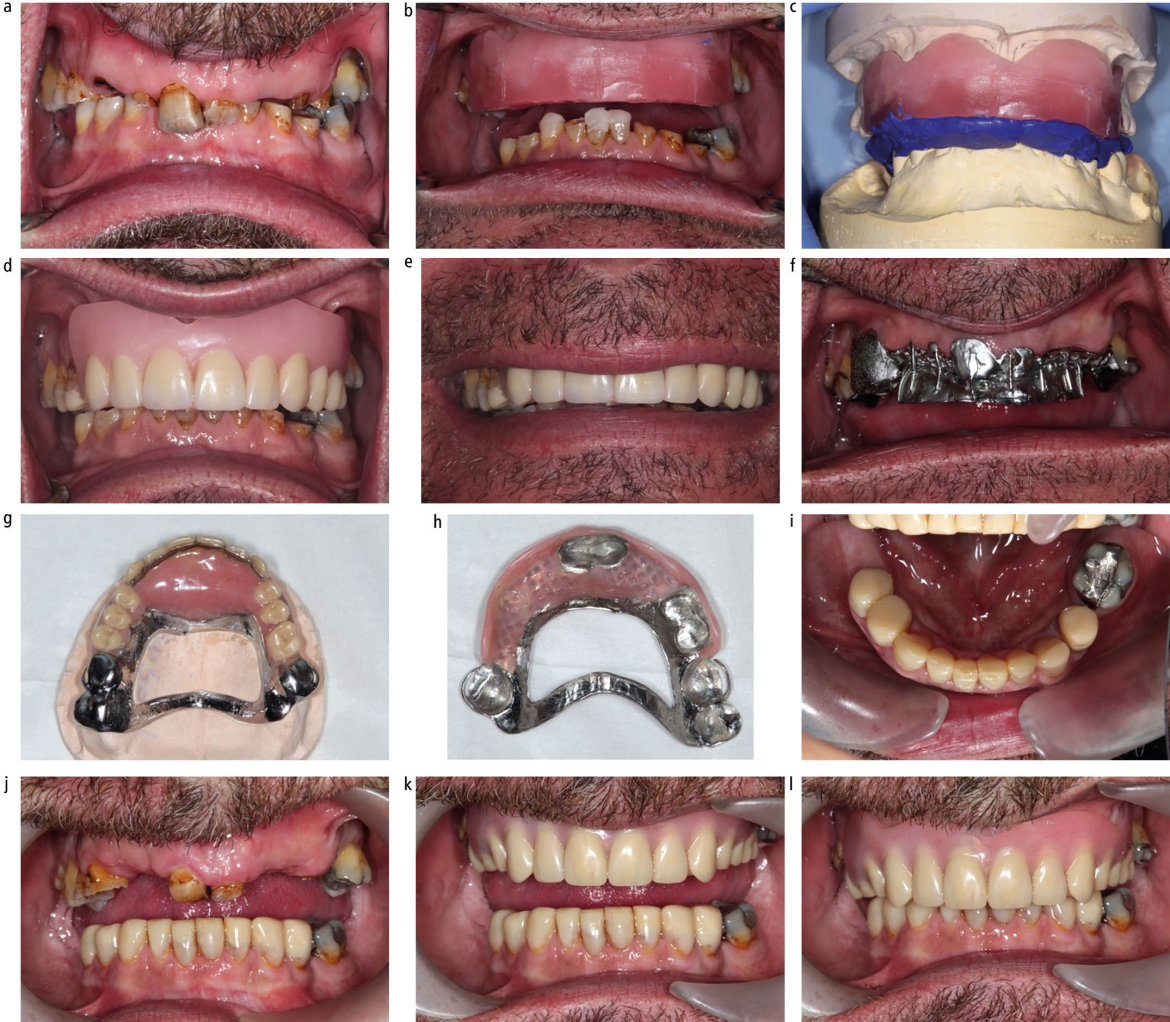
Case photographs for a separate arch fixed-removable case with severe erosive and attritional tooth wear. a) Pre-operative retracted view. b, c) Jaw registration completed with maxillary wax rim, temporary composite mock-up of 41, 43, 31 at desired OVD, followed by segmental removal of composite for silicone registration material. d, e) Wax-tooth try-in to verify tooth position and lip support. f) Metal framework try-in of maxillary partial denture. Retention tags to support prosthetic teeth. g, h) Completed maxillary denture ready to fit. Note acrylic anterior bite platform, which was essential to provide anterior tooth contact in this Class II division 1 incisor relationship. i, j) Completed mandibular direct composite build-up restorations. k, l) Completion photographs, with maxillary denture in situ

**Fig. 10:**
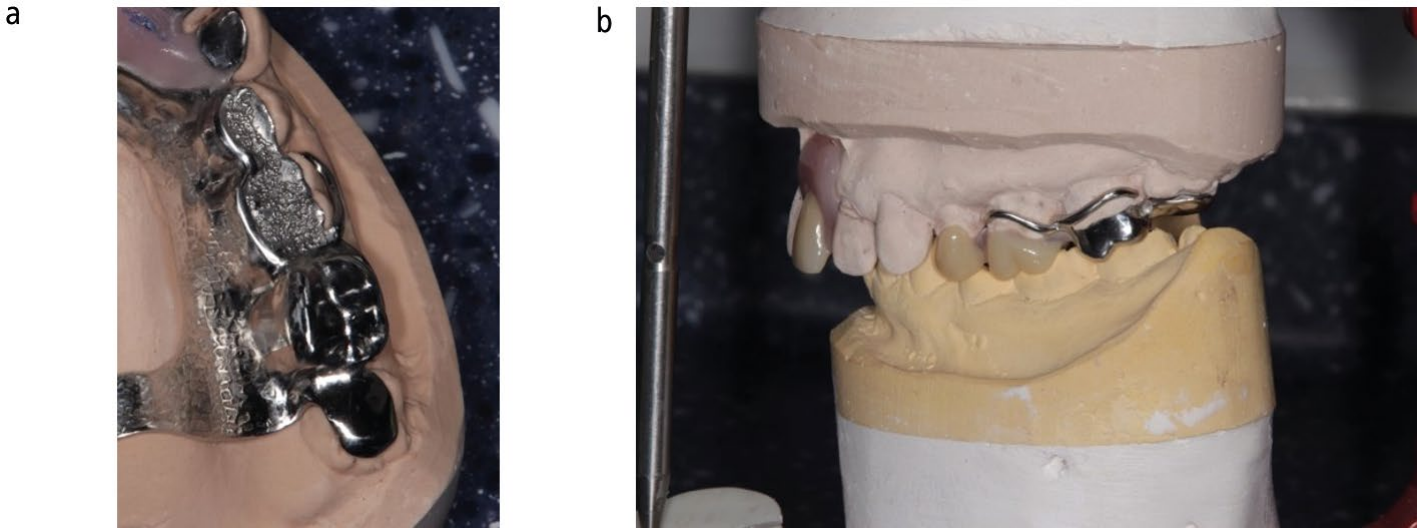
a, b) Metal occlusal onlay rests 25, 26. Hybrid metal-acrylic occlusal onlay rests 27, 28 in more aesthetic zone and where increased interocclusal space

When increasing the vertical dimension, it is sometimes necessary to provide an anterior bite platform to ensure functional anterior tooth contact, especially in Class I and II incisor relationships (Fig. 9g). It should be remembered that when increasing the vertical dimension, the overjet is also increased and this sometimes comes with loss of anterior tooth contact. The choice of material for this is clinician- and patient-driven. Acrylic bite platforms, for the majority, are acceptable and benefit from ease of adjustment at the chairside and reduced technical challenges. However, if there is insufficient interocclusal space for acrylic, or the patient requires a more robust bite platform, then metal may be a more predictable option. This principle also applies to the choice of material for occlusal onlays (Fig. 10). Where space is limited, metal occlusal onlays on posterior teeth are preferred, since acrylic requires a minimum of 2 mm interocclusal space to be sufficiently rigid, versus 1 mm cobalt-chrome. Acrylic onlays are more prone to fracture and should be avoided in a patient with parafunction. Metal is more resistant to wear than acrylic and will hold the newly established vertical dimension more predictably. It is the only material of choice for a parafunctioning patient.

Overlay prostheses often suffer from lack of bucco-lingual space when balancing lip support and functional tooth positions. For this reason, the space required for the metal framework to cover the natural teeth, followed by the prosthetic teeth overlying this, is often challenging and the prosthetic teeth are at risk of being unsupported and lacking retention. To counteract this issue, retention tags or loops (Fig. 9f) within the metal framework can be of benefit. However, in some circumstances, the space allowances do not accommodate even these features and they need to be omitted. 4-methacryloxyethyl trimellitate anhydride metal adhesive monomer offers predictable bonding of acrylic to cobalt-chrome frameworks and reduces the need for micromechanical retentive features.

Major connectors for maxillary dentures can be ring connectors, plates or mid-palatal straps. All designs are rigid and avoid the risk of deformation under excessive occlusal loads. For mandibular dentures, lingual plates provide the effect of metal backings against compromised anterior teeth, even when these teeth are restored with direct composite restorations (Fig. 2).

## Clinical stages: provision of only removable dentures

Table 1 describes the treatment sequence of such cases.

**Table 1:** Treatment sequence for sole removable chrome denture cases in the management of tooth wear

No.	Step
1	Primary impressions
2	Tooth preparation (if required) and master impressions
3	Jaw registration at desired occlusal vertical dimension
4	Wax-tooth try-in
5	Metal framework try-in
6	Definitive metal-wax-tooth try-in
7	Fit

The authors favour alginate or a hybrid of light/medium-viscosity silicone in a custom-spaced (2-3 mm) tray for the master impression.

A registration of the desired occlusal vertical dimension is performed by using a wax rim over the teeth to be used as overdenture abutments and the patient is carefully guided into centric relation. Registration pastes allow accurate recording of the jaw relationships and the wax rims should be checked back on the master models to establish reproducibility in the laboratory.

Prior to metal framework construction, the vertical dimension, occlusion, lip support, tooth position and mould should be verified with a wax-tooth try-in. Once these features have been established and accepted, the metal framework can be reverse-engineered by taking a putty index of the tooth position and locating this back to the master model. In this way, features such as metal backings, retention tags and loops can be accurately positioned to the final tooth position.

After the metal work has been tried in, a penultimate wax/tooth/metal try-in appointment is recommended. There may be 'greying out' of the denture teeth due to the metal backing shining through and the patient should have an opportunity to check this and request a more opaque shade for their acrylic teeth, if needed.

## Clinical stages: combined fixed and removable cases

Teeth which have been less severely affected by wear may be predictably restored with fixed restorations, while there might be a need to replace missing or severely worn teeth with a removable denture. This section is further subdivided in to:

- Provision of fixed and removable restorations in the same arch
- Provision of a removable denture in one arch and fixed restorations in the opposing arch.

### Provision of fixed and removable restorations in the same arch

When providing a combination of fixed restorations and removable dentures in the same arch, it is impossible to avoid a transitional acrylic denture phase. This is due to the inability to accurately predict the final contour and margins of fixed restorations while constructing a precisely fitting metal removable framework. For this reason, a transitional acrylic denture should be made to be delivered at the time of fit of fixed restorations, which can be altered easily at the chairside, to provide (often) posterior occlusal support. See Table 2 for treatment delivery stages.

**Table 2:** Treatment sequence for same arch fixed and removable prostheses in the management of tooth wear

No.	Step
1	Primary impressions
2	Master impressions in a special tray to optimise the denture bearing anatomy
3	Jaw registration at desired increase in OVD
4	Aesthetic preview^14^^,^^15^of fixed restorations (using stent over wax-up and bis-acrylic material) and removable dentures (as a wax-tooth try-in). Ensure OVD and occlusal contacts are correct
5	Deliver fixed restorations (either direct or indirect) and interim acrylic partial dentures. Likely to be delivered over multiple appointments. Dentures to be fitted at last appointment
6	Construct new cobalt-chrome removable partial dentures if required at new OVD set by fixed restorations

**Fig. 11:**
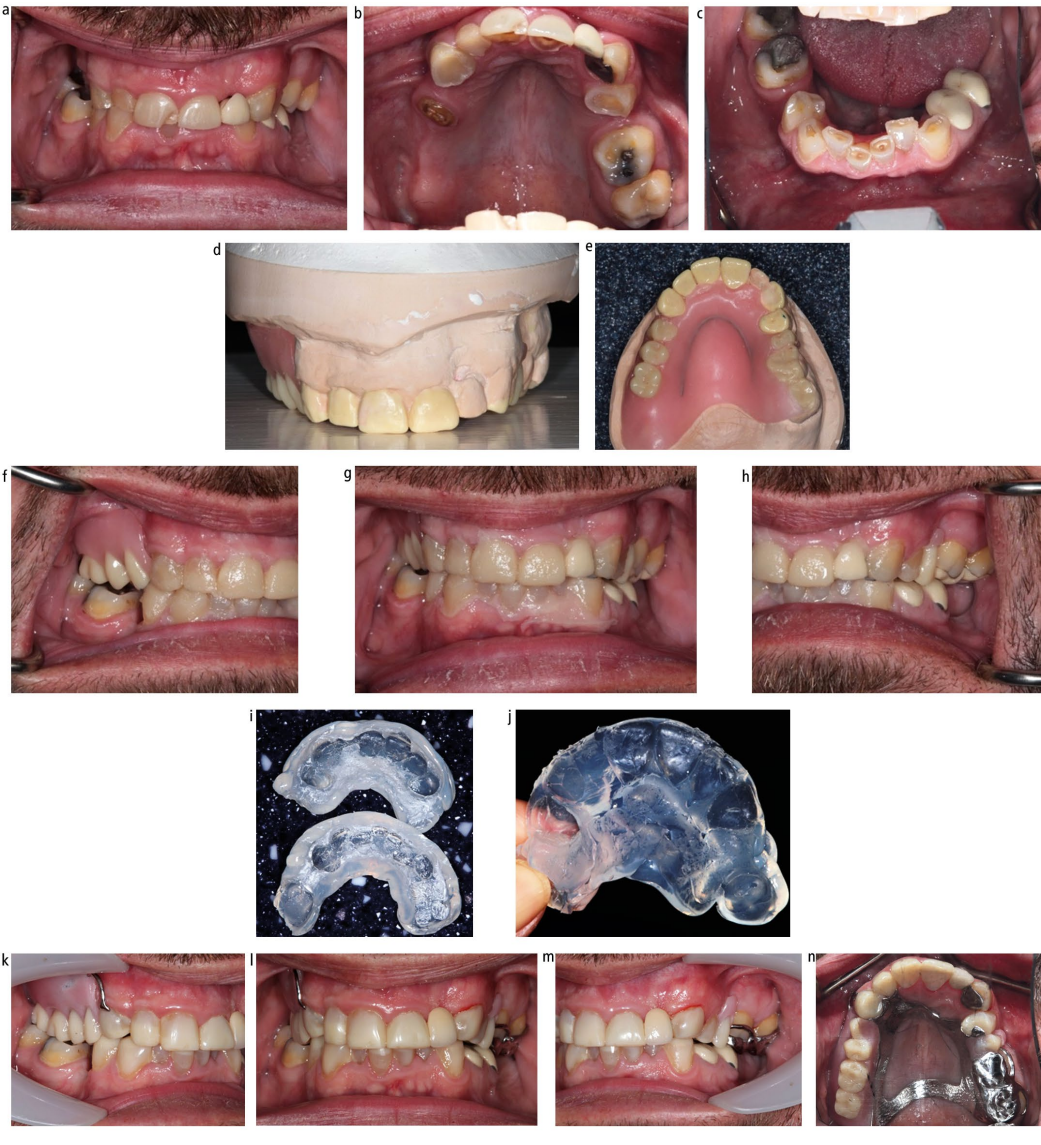
Moderate generalised erosive tooth wear treated with same arch maxillary direct composite build-ups, resin-bonded bridge 22, 23 to replace 22 and definitive cobalt-chrome partial denture, and mandibular direct composite build-up restorations. The patient required an interim phase with acrylic partial denture before the definitive cobalt-chrome denture was constructed. a, b, c) Pre-operative views demonstrating erosive tooth wear in both arches, chipped existing composite restorations and failing Resin bonded bridge 22,23. d, e) Diagnostic wax-ups and maxillary partial denture wax-tooth try-in made on articulated study models mounted at the desired OVD on a semi-adjustable articulator. Note how the wax-tooth try-in has acrylic onlay rests on 26, 27 a to provide tooth support at the increased OVD. f, g, h) Aesthetic preview appointment with temporary crown and bridge material (Integrity, Dentsply Sirona) on teeth to have composite restorations, and wax-tooth try-in maxillary partial denture. i) Addition cured silicone (Memosil 2, Kulzer) full coverage indices made from diagnostic wax-ups. j) Palatal addition cured silicone guide used for direct composite build-up restorations, based on the confirmed diagnostic wax-up. k, l, m, n) Post-operative photographs following provision of definitive maxillary cobalt-chrome partial denture. The completed restorations were direct composite build-up restorations on 11, 12, 13, 21, 23, 31, ,32, 33, 41, 42, 43, resin-bonded bridge 22, 23, maxillary partial cobalt-chrome denture

In order to time the clinical events accurately, a preview appointment is required to try in both the planned fixed restorations and a wax-tooth try-in of the denture (Fig. 11f-h). This allows confirmation of the planned increase in vertical dimension and tooth display. The denture can then be processed to fit at the delivery of the fixed restorations to avoid delay in receiving posterior support for the new anterior restorations. Following an adaptation phase, which is often short, the transitional denture can then be replaced by a definitive cobalt-chrome denture, conforming to the newly established OVD.

### Provision of a removable denture in one arch and fixed restorations in the opposing arch

For separate arch fixed/removable cases, the clinician can again work the patient through to an aesthetic preview stage to trial the fixed and removable restorations.^17^^,^^18^ However, they can omit the need for a transitional acrylic denture. This means that a cobalt-chrome denture can be made as the first denture. See Table 3 for the treatment sequence and Figure 9.

**Table 3:** Treatment delivery sequence for separate arch fixed and removable prostheses in the management of tooth wear

No.	Step
1	Primary impressions
2	Master impressions
3	Jaw registration at desired OVD
4	Aesthetic preview try-in of fixed restorations^14^^,^^15^(using stent over wax-up and bis-acrylic material) and removable dentures (as a wax-tooth try-in)
5	Removable metal framework try-in
6	Definitive metal-wax-tooth try-in
7	Fit removable denture
8	Deliver fixed restorations (either direct or indirect)

The authors tend to provide the removable denture first in order to maximise the adaptation time and to assess patient tolerance to an increased vertical dimension. Use of acrylic bite platforms are imperative in these cases to enable adjustment of the acrylic component of the denture, rather than the fixed restorations, when harmonising the occlusion at the end of treatment.

## Maintenance issues

As with all complex restorative dentistry, maintenance is key for the survival and success of restorations. Multiple studies have shown an increased incidence of complications and loss of overdenture abutment teeth if they are not regularly reviewed and maintained.^15^^,^^19^^,^^20^^,^^21^^,^^22^ Frequent complications of overdenture abutments are periodontal disease, caries and periapical pathology. Despite this, high abutment tooth survival rates have been shown over several years.^19^^,^^20^^,^^21^ Factors associated with increased abutment tooth loss are infrequent recalls (less than annually), infrequent use of high fluoride toothpaste (5,000 ppm fluoride),^19^ 24 hour wear of dentures^22^ and medically compromised patients. Therefore, the authors suggest the prescription of high fluoride toothpaste for use twice daily for brushing and once daily inside of the denture, removal of the prostheses at night, and three-monthly recalls, with focused oral hygiene instruction for all overdenture patients.

The incidence of endodontic failure of overdenture abutment teeth is generally low.^15^ Inadequate oral hygiene resulting in caries and restoration failure is the most common cause for endodontic complications in these patients. Endodontic therapy to teeth with short clinical crown heights can be challenging and sometimes, extraction of the teeth is more predictable. Annual dentine bonding sealant of overdenture abutment teeth may reduce endodontic complications with vital abutments.^15^

Overdenture abutment teeth in the maxilla opposed by natural teeth are at an increased risk of vertical root fractures.^19^ This is likely especially true while the dentures are not in place at night. For this reason, and to protect concurrent fixed restorations, provision of nocturnal splints are advised. The choice of splint is dependent on the aetiology of tooth wear, with most patients suiting a soft bite raising appliance, but patients with severe parafunction may benefit from a heat-cured acrylic splint.

Overdentures frequently require maintenance, such as base adjustments and relines.^23^ In tooth wear patients, the most frequent failures are occlusal surface fractures.^24^ In order to reduce this, the authors recommend the use of metal on the occlusal surfaces and minimal acrylic flanges to facilitate future chairside relines should overdenture abutments require extraction in the future.

## Conclusion

A severely worn dentition is difficult to treat. If there are already a number of missing teeth and the remaining dentition is very worn, with only sclerotic dentine remaining and short roots, then an overdenture/overlay denture may represent a more predictable, biologically conservative and quick means of rehabilitation. Retention of roots/teeth maintains proprioception, alveolar bone and soft tissue undercuts. These can be used to optimise retention by prescribing minimal, scalloped flanges, 1 mm above any soft tissue undercuts around any remaining roots. Chrome-based frameworks are the material of choice, especially in cases where parafunction is an issue or interocclusal space is minimal. Metal onlays can be used on posterior teeth to limit maintenance issues and backings should be prescribed around any teeth of guarded prognosis. Patients should be prescribed a mouthguard for nocturnal wear, as they will help to preserve the fit of the overlay/onlay denture in the long term. Patients should be advised to see their practitioners on a 3-4-monthly basis to ensure that they are maintaining optimal plaque control around the denture and should be advised to load their prosthesis with fluoride toothpaste to minimise the development of future carious lesions.
